# Prenatal Fetal Growth Restriction and Cardiomyopathy Associated With an Unbalanced der(15)t(7;15): A Case Report

**DOI:** 10.1002/ccr3.73104

**Published:** 2026-07-14

**Authors:** Yoko Nagayasu, Nagisa Ishikawa, Seiichiro Nao, Atsushi Yoshida, Hiroshi Ohta, Misa Nunode, Mayumi Nakamura, Takumi Sano, Masae Yoo, Yutaka Odanaka, Daisuke Fujita, Tomohito Tanaka

**Affiliations:** ^1^ Department of Obstetrics and Gynecology Osaka Medical and Pharmaceutical University Osaka Japan; ^2^ Kanki Obstetrics and Gynecology Clinic Osaka Japan; ^3^ Department of Pediatrics Osaka Medical and Pharmaceutical University Osaka Japan

**Keywords:** 15q26 deletion, chromosomal translocation, fetal cardiomyopathy, fetal growth restriction(FGR), preimplantation genetic testing for structural rearrangements (PGT‐SR)

## Abstract

Severe fetal growth restriction with cardiomyopathy may indicate an underlying chromosomal abnormality. Genetic evaluation is essential for accurate counseling and management, and preimplantation genetic testing for structural rearrangements (PGT‐SR) may be considered for future reproductive planning.

## Graphical abstract

Summary of the key chromosomal findings identified by conventional chromosomal analysis and chromosomal microarray analysis.

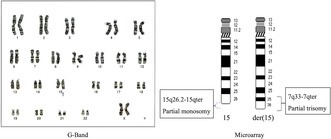

## 1 Introduction

Fetal cardiomyopathy is a rare but potentially life‐threatening condition, with an estimated prenatal prevalence of 0.01% to 0.04%. Its causes are diverse, including genetic, metabolic, and structural factors. Among these, left ventricular noncompaction cardiomyopathy (LVNC) and dilated cardiomyopathy may present prenatally with cardiomegaly and impaired cardiac function. Chromosomal abnormalities are associated with both fetal growth restriction (FGR) and congenital heart disease (CHD). However, the role of structural chromosomal rearrangements is less well described.

Deletions of chromosome 15q26 are associated with FGR and prenatal cardiomyopathy, often related to haploinsufficiency of the IGF1R gene (reduced gene dosage affecting fetal growth).

Here, we present a rare case of severe FGR and prenatal cardiomyopathy associated with an unbalanced translocation, 46,XX,der(15)t(7;15)(q33;q26.2), inherited from a maternal balanced translocation.

This case is unique because it combines a rare chromosomal rearrangement with prenatal cardiomyopathy and severe growth restriction. We present this case to raise awareness of this genetic cause and to emphasize the importance of early genetic testing and appropriate family counseling.

## 2 Case History

A 31‐year‐old primigravida with no significant medical history conceived spontaneously and received routine prenatal care at a local clinic. She had no history of previous pregnancies or miscarriages. Her family history was unremarkable, except for a younger brother with a learning disability. Routine prenatal screening tests were performed at the referring clinic, with no abnormalities reported.

At 24 weeks of gestation, FGR was noted, with an estimated fetal weight (EFW) of −2.5 standard deviations (SD), and cardiomegaly was suspected.

**FIGURE 1:**
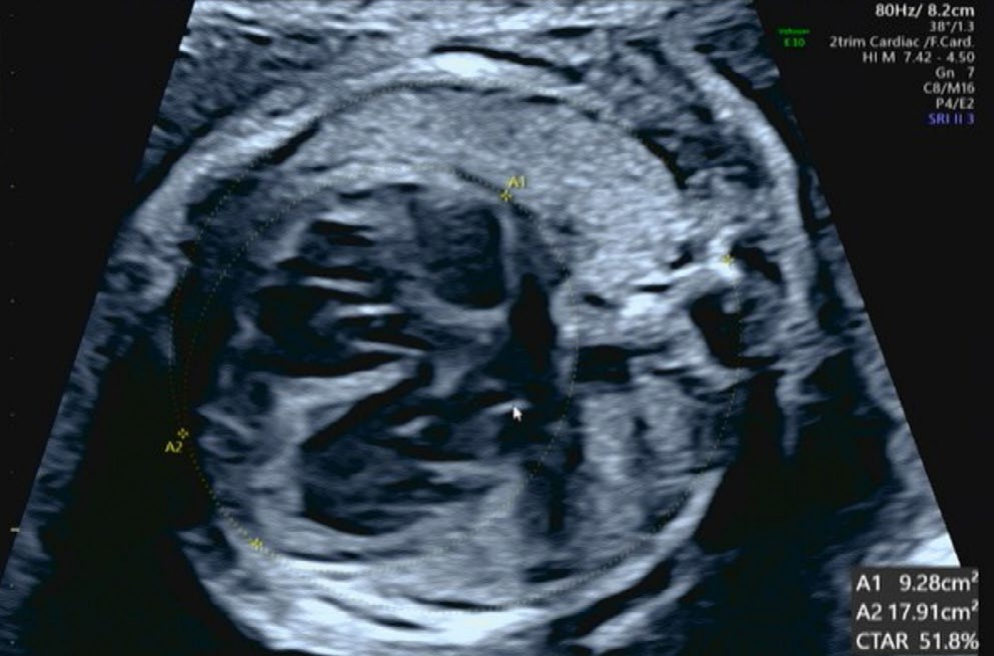
Fetal ultrasound at 29 weeks showing marked cardiomegaly and left ventricular noncompaction.

**FIGURE 2:**
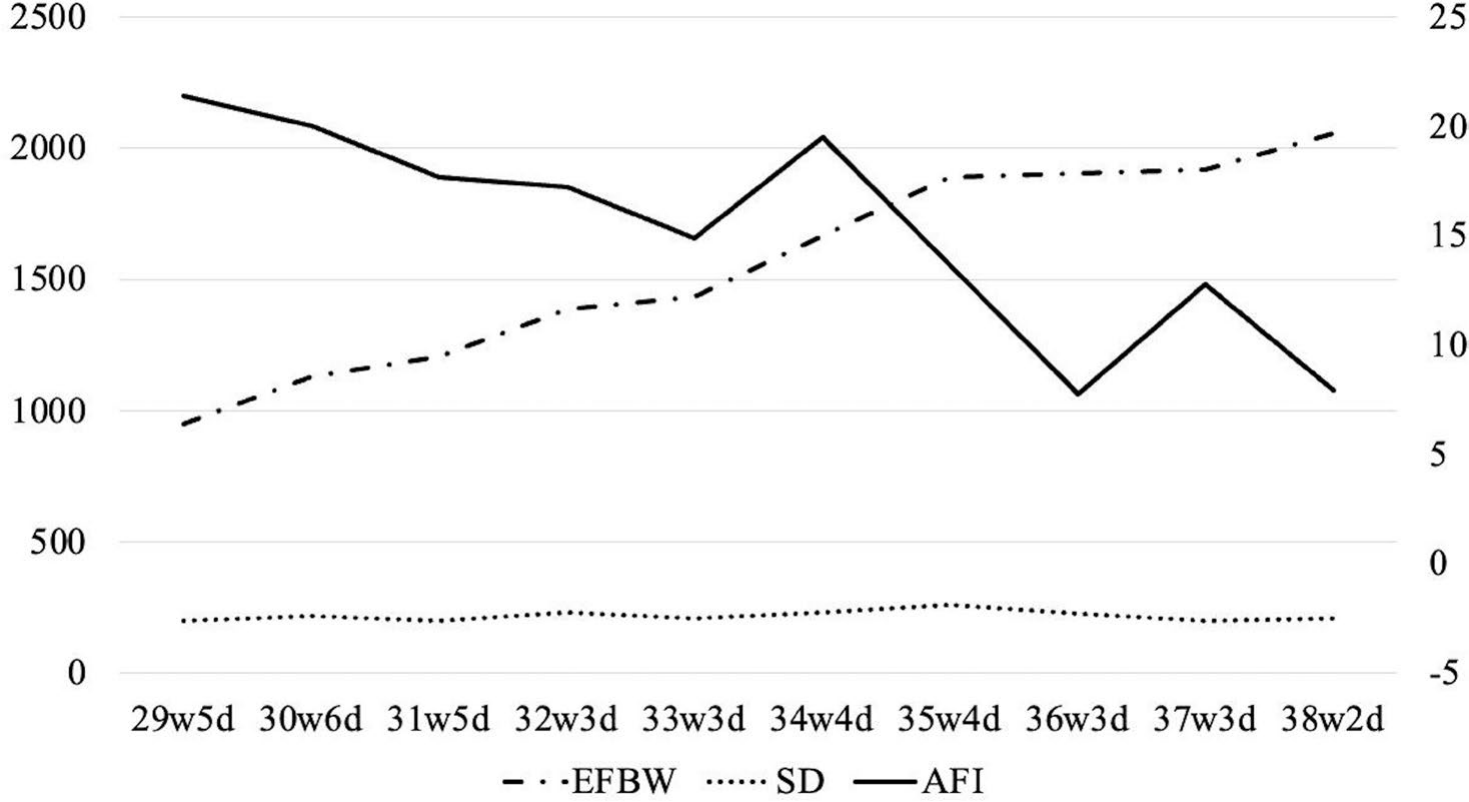
Serial changes in fetal growth and amniotic fluid volume. X‐axis: Gestational age (weeks) Left Y‐axis: Estimated fetal weight (g) Right Y‐axis: Amniotic fluid index (cm), Estimated fetal weight (SD).

At 29 weeks and 5 days, she was referred to our perinatal center for further evaluation. Ultrasound examination revealed an EFW of 950 g (−2.6 SD), an amniotic fluid index of 21.4 cm, and a cardiothoracic area ratio (CTAR) of 51% (Figure 1). Detailed fetal echocardiography demonstrated left ventricular dilation with trabeculated myocardium suggestive of noncompaction cardiomyopathy, a 4 mm muscular ventricular septal defect, and possible coarctation of the aorta. The umbilical artery Doppler flow showed normal resistance, and no signs of hydrops or placental insufficiency were present. The cardiovascular profile score (CVPS) was 8/10, reflecting mild cardiomegaly (CTAR 51%) and mildly reduced cardiac function, without evidence of fetal hydrops, abnormal Doppler findings, or valvular regurgitation. No extracardiac anomalies were detected. Figure 2 illustrates the serial changes in estimated fetal weight and amniotic fluid volume during pregnancy.

## 3 Diagnostic Investigations and Management

Given the cardiac findings, several differential diagnoses were considered, including metabolic disorders, viral myocarditis, and syndromic or chromosomal abnormalities. Although invasive prenatal testing was recommended to evaluate chromosomal abnormalities, the parents declined due to concerns about the risks of amniocentesis, such as premature rupture of membranes. The parents were counseled regarding the potential benefits and risks of prenatal genetic testing, including its diagnostic value and possible implications for clinical management and prognosis. Serial ultrasounds showed persistent growth restriction without significant cardiac deterioration. After multidisciplinary consultation with pediatric cardiologists and neonatologists, a planned cesarean section was scheduled at 38 weeks and 3 days of gestation, given the risk of hemodynamic instability after delivery associated with cardiomyopathy and the potential need for urgent intervention to enable immediate postnatal cardiac evaluation and management.

A female infant was delivered under spinal‐epidural anesthesia. Birth weight was 1688 g (< −2.5 SD), with Apgar scores of 7 and 8 at 1 and 5 min, respectively. Umbilical artery blood gas analysis revealed a pH of 7.351 and base excess of −1 mmol/L. Postnatal echocardiography confirmed the diagnosis of left ventricular noncompaction cardiomyopathy (LVNC) and ventricular septal defect (VSD). No outflow obstruction was noted at that time. The infant was managed with careful hemodynamic monitoring in the neonatal intensive care unit. Medical management was initiated for heart failure symptoms as needed.

At 20 days of age, the infant underwent pulmonary artery banding due to progressive volume overload. At 55 days, she required pericardial drainage for acute cardiac tamponade.

## 4 Postnatal Findings and Genetic Diagnosis

Following these interventions, her condition stabilized, and she was discharged in stable condition at 106 days of age.

**FIGURE 3:**
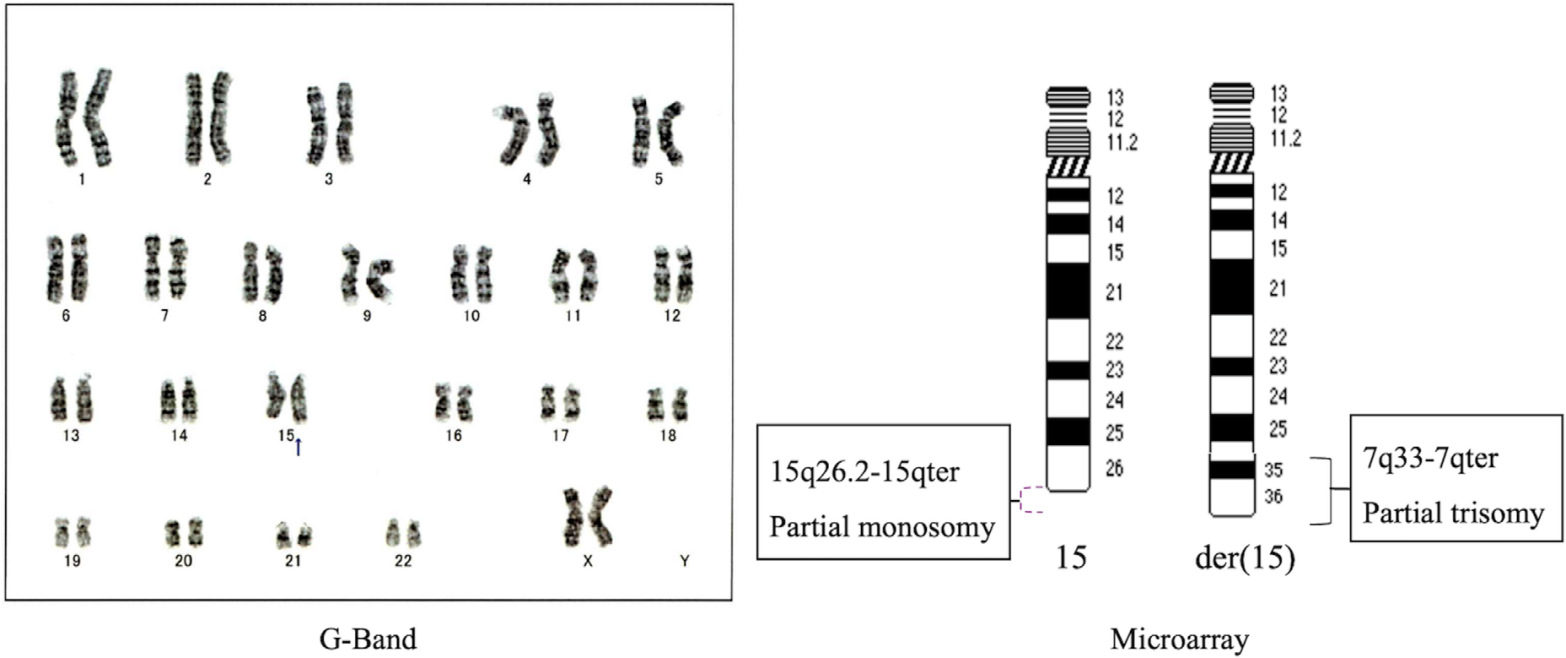
Postnatal chromosomal and microarray findings of the proband.

Postnatal genetic evaluation included karyotyping and chromosomal microarray analysis. Chromosomal microarray analysis was performed using a commercially available platform (SRL, Tokyo, Japan), with a resolution sufficient to detect submicroscopic copy number variations. Conventional karyotyping revealed an unbalanced translocation: 46,XX,der(15)t(7;15)(q33;q26.2) (Figure 3). The chromosomal microarray detected a deletion at 15q26.1–qter encompassing multiple growth‐related and cardiac development genes, including *IGF1R* and *NR2F2*. IGF1R plays a critical role in fetal growth, while NR2F2 is involved in cardiac development, particularly in cardiac morphogenesis. Detailed genomic coordinates and exact deletion size were not available.

**FIGURE 4:**
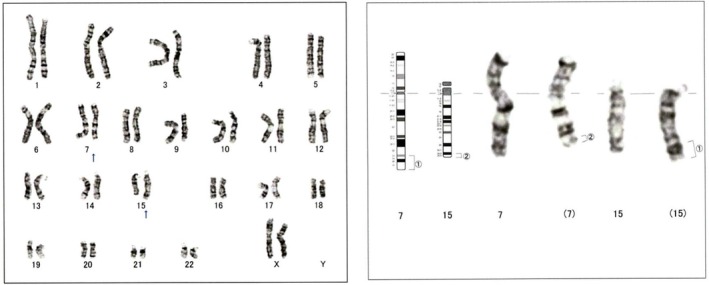
Karyotype of the mother showing a balanced reciprocal translocation between chromosomes 7 and 15: 46,XX,t(7;15)(q33;q26.2).

Parental chromosomal testing demonstrated a balanced translocation in the mother (Figure 4): 46,XX,t(7;15)(q33;q26.2), while the father's karyotype was normal. Genetic counseling was provided to the family, and the option of preimplantation genetic testing for structural rearrangements (PGT‐SR) was discussed for future pregnancies.

## 5 Discussion

Fetal cardiomyopathy is a rare condition with a prenatal prevalence of approximately 0.01%–0.04% [1, 2]. Left ventricular noncompaction cardiomyopathy (LVNC) is characterized by excessive trabeculation and may be difficult to diagnose prenatally due to limited hemodynamic findings [3, 4].

In this case, the fetus had LVNC and a ventricular septal defect (VSD), along with severe fetal growth restriction (FGR). This combination strongly suggested an underlying genetic cause, which was confirmed by postnatal genetic testing. Congenital heart disease is more common in fetuses with FGR than in those with normal growth.

The genetic findings demonstrated an unbalanced translocation involving chromosomes 7 and 15, resulting in a 15q26 deletion and partial 7q duplication. The 15q26 region includes IGF1R, which plays a key role in fetal growth, and its deletion likely explains the severe FGR observed [5, 6, 7, 8]. In addition, NR2F2, also located in this region, is involved in cardiac development and may contribute to the cardiac phenotype [9, 10, 11].

Previous studies have reported that 15q26 deletions are associated with congenital heart defects in up to 50%–70% of cases [6, 12]. However, cardiomyopathy, particularly LVNC, has rarely been described, suggesting that this case represents an uncommon presentation. The specific role of NR2F2 in myocardial compaction remains unclear, and its association with LVNC has not been well established.

The contribution of the 7q duplication remains uncertain, but it may have had a modifying effect on the phenotype. Distal 7q duplications have been associated with growth restriction and congenital heart defects [13, 14]. Therefore, the cardiac findings in this case may primarily be explained by the 15q26 deletion, with a possible additional contribution from the 7q duplication.

Clinically, this case highlights the importance of considering chromosomal abnormalities in fetuses presenting with both FGR and cardiac findings. Early genetic evaluation is essential for accurate diagnosis and counseling.

In addition, parental balanced translocation has important implications for recurrence risk. Preimplantation genetic testing for structural rearrangements (PGT‐SR) may be considered in future pregnancies [15, 16].

This case also emphasizes the value of fetal echocardiography and postnatal genetic testing in identifying underlying genetic causes and guiding clinical management.

## 6 Conclusion

This case highlights the importance of considering chromosomal abnormalities in fetuses presenting with both growth restriction and cardiac findings. Genetic diagnosis is essential for accurate counseling and clinical management. PGT‐SR may be considered as an option for future reproductive planning.

## Author Contributions

Y.N. conceptualized the study, performed fetal ultrasound examinations, collected clinical data, and drafted the manuscript. N.I., S.N., A.Y., H.O., M.N., M.N., M.Y., Y.O. and T.S. contributed to clinical management and data interpretation. D.F. and T.T. supervised pediatric and genetic evaluations. All authors reviewed and approved the final manuscript.

## Funding

The authors have nothing to report.

## Ethics Statement

Written informed consent for publication of clinical details and images was obtained from the patient's parents in accordance with Wiley's patient privacy and ethics guidelines.

## Conflicts of Interest

The authors declare no conflicts of interest.

## Data Availability

Data sharing is not applicable to this article as no datasets were generated or analyzed beyond the clinical information contained within the case report.
